# People Living with HIV Eligibility in Canadian Cancer Clinical Trials

**DOI:** 10.3390/curroncol33020102

**Published:** 2026-02-05

**Authors:** Maria F. Comelles, Santiago Perez-Patrigeon, Tessa Senneker, Anna Johnson, Lisa K. Hicks, Lynda Balneaves, Bingshu E. Chen, Annette E. Hay

**Affiliations:** 1 Canadian Cancer Trials Group, Queen’s University, Kingston, ON K7L 3N6, Canada; ajohnson@ctg.queensu.ca (A.J.); bechen@ctg.queensu.ca (B.E.C.); ahay@ctg.queensu.ca (A.E.H.); 2 Kingston General Hospital, Kingston Health Sciences Centre, Queen’s University, Kingston, ON K7L 2V7, Canada; santiago.perezpatrigeon@kingstonhsc.ca (S.P.-P.); tessa.senneker@kingstonhsc.ca (T.S.); 3 Division of Hematology, Li Ka Shing Knowledge Institute, St. Michael’s Hospital, Unity Health Toronto, Toronto, ON M5B 1W8, Canada; lisak.hicks@unityhealth.to; 4 College of Nursing, University of Manitoba, Winnipeg, MB R3T 2N2, Canada; lynda.balneaves@umanitoba.ca

**Keywords:** health equity, clinical trials as a topic, medical oncology, HIV Infections, eligibility determination

## Abstract

**Background:** People living with HIV (PLWH) have historically been excluded from cancer clinical trials, prompting the 2017 ASCO–Friends of Cancer Research recommendations to limit unjustified exclusions and promote equitable access. This study evaluated how Canadian Cancer Trials Group (CCTG) protocols align with these recommendations. **Methods:** We conducted a cross-sectional review of CCTG active trial protocols, abstracting HIV eligibility language and trial characteristics, and assessing associations using Chi-square tests. **Results:** Of 136 trials activated between 1999 and 2025, 81.6% involved solid tumors, 63.2% systemic therapy interventions, and 61.5% phase III designs. PLWH were included, not mentioned or excluded in 49/136 (36%), 55/136 (40.4%) and 32/136 (23.5%) respectively, with justification in 7/32 (21.9%). In multivariable analyses, exclusion was more likely in trials of immune checkpoint blockade therapy (*p* = 0.039) and those with industry support (*p* = 0.014). Adjusted models showed that both industry sponsorship and immune checkpoint blockade independently reduced HIV trial inclusion. **Conclusions:** Most CCTG-associated trials were inclusive or neutral toward PLWH; however, a proportion still excluded them, often without justification. This first national assessment evaluating adoption of ASCO Friends of Cancer Research–HIV guidance establishes a Canadian benchmark for equitable trial design and future research as both cancer and HIV therapies continue to evolve.

## Simple Summary

People living with HIV have historically been excluded from many cancer clinical trials, limiting their access to new therapies and reducing the generalizability of research. Recent recommendations from professional societies encourage broader inclusion. The extent to which Canadian trials follow these guidelines has not been described. In this study, we reviewed 136 cancer trials conducted with the Canadian Cancer Trials Group and found that most did not restrict participation of people living with HIV. However, some trial protocols still excluded them without justification. Trials using immune checkpoint inhibitor therapies, and those run with industry support, were more likely to exclude people living with HIV. Greater inclusion could help ensure equitable access to research, guide policy updates, and support more representative cancer studies in Canada.

## 1. Introduction

As life expectancy for people living with HIV (PLWH) has improved with antiretroviral therapy (ART), a rising number are growing older while managing the infection. Among this population, cancer has become increasingly common, occurring at a greater incidence than in the general population [1]. This elevated cancer burden is attributed to HIV-related immune impairment, a higher prevalence of risk factors such as tobacco use and oncogenic viral co-infections, and aging. While ART led to a marked decline in cancer incidence, malignancies continue to be among the leading causes of death in PLWH in North America [2].

In Canada, an estimated 65,270 people were living with HIV by the end of 2022 [3]. A matched population-based study in Ontario (1996–2020) found that PLWH had nearly eight times higher risk of infection-related cancers than those without HIV. The risk of developing such cancers by age 65 dropped from 19% in the early ART era (1996–2011) to 10% in the contemporary ART era (2012–2020), mainly due to declines in virus-related cancers. However, the risk of some types of anal and liver cancers remained elevated, with excess cancer risk more pronounced at younger ages [4].

Despite the predominance of cancer among PLWH, they have been historically underrepresented in cancer clinical trials. A study of lung cancer clinical trials demonstrated that PLWH were excluded with little scientific justification [5]. In a survey of United States (US)-based medical and radiation oncologists, 69% of the 500 respondents felt that available cancer management guidelines were insufficient for the care of PLWH [6]. A review of 56 lymphoma trials in the United Kingdom found that 70% of interventional studies excluded PLWH; scientific justification for exclusion was identified in only one trial [7]. The American Society of Clinical Oncology (ASCO) reviewed 46 New Drug Applications that led to initial US Food and Drug Administration (FDA) approval from 2011 to 2015, revealing that exclusion of PLWH remained common in most studies of novel cancer agents. Therefore, the FDA [8] and ASCO–Friends of Cancer Research (AFCR-HIV) developed recommendations to enable appropriate inclusion of PLWH in cancer clinical trials. These recommendations include (i) broad eligibility for patients with CD4+ counts ≥ 350 cells/µL; (ii) inclusion of those with no or remote opportunistic infections; (iii) concurrent ART with specified timing and viral control criteria; (iv) careful management of drug–drug interactions and overlapping toxicities; (v) flexibility in ART use when cancer therapy must be prioritized; and (vi) rational exclusion of specific ART agents when necessary [9].

In the early antiretroviral era, concerns included drug interactions and overlapping toxicities between ART and cancer therapies, and increased infection-related mortality in the setting of advanced HIV-related immune suppression. Currently available ART improves immune control among PLWH, has a lower potential for drug interactions, and reduces overlapping toxicities with cancer therapies [10]. Modernizing eligibility criteria to appropriately include PLWH in cancer clinical trials is critical to advance understanding of cancer management in this population. Multiple National Cancer Institute (NCI)-US-sponsored studies have shown that their inclusion is both feasible and safe, and HIV infection alone should no longer serve as an exclusion criterion [11,12,13,14,15]. Inclusion of PLWH in cancer clinical trials may increase rates of accrual and study completion, provide more equitable access to emerging therapeutics, and lead to more generalizable results.

To our knowledge, no Canadian institution has evaluated the inclusion of PLWH in national cancer clinical trials, nor has it examined how the AFCR-HIV recommendations have influenced eligibility criteria or trial inclusion practices. The Canadian Cancer Trials Group (CCTG) is a national cooperative oncology network that designs and conducts cancer therapy, supportive care, and prevention trials across Canada. Established in 1980, it includes over 85 member institutions and collaborates with partners in more than 40 countries. Headquartered in Kingston, Ontario, CCTG leads and sponsors both domestic and international studies through its Operations and Statistics Centre. We retrospectively reviewed CCTG studies to evaluate alignment with the AFCR-HIV recommendations.

## 2. Methods

We conducted a retrospective, cross-sectional analysis following the EQUATOR Reporting Guidelines [16]. The Queen’s University Health Sciences and Affiliated Teaching Hospitals Research Ethics Board granted an exemption under the Tri-Council Policy Statement: Ethical Conduct for Research Involving Humans (TCPS 2, Article 2.5). The multidisciplinary team—with expertise in hematology, infectious diseases, HIV-related malignancies, clinical trial design, biostatistics, pharmacy, equity, diversity, inclusion and community engagement—provided clinical and methodological oversight.

Between 1 April 2025 and 16 May, we reviewed CCTG active clinical trial protocols that were open to accrual and in follow-up using data housed at the Operations and Statistics Centre at Queen’s University. Sub-studies were excluded because they typically duplicate the eligibility framework of their parent protocol and therefore do not provide independent or additional HIV-related criteria. Permanently closed trials were also excluded, as full protocol documents are often unavailable, limiting the ability to reliably extract and evaluate HIV-specific eligibility requirements. Supplemental information was obtained from the trial archives, which include related publications, abstracts, and presentations.

Data extraction was performed by F Comelles with input from A Hay and entered into a standardized Excel database capturing cancer type, trial phase, activation year, industry funding, lead group, and intervention type. HIV eligibility was classified as: (i) eligible—explicitly stated as eligible; (ii) ineligible—explicitly excluded; or (iii) not mentioned—no reference to HIV status. Where available, we recorded HIV-specific criteria for inclusion (e.g., CD4 count, ART use, viral load, or opportunistic infections) and stated reasons for ineligibility.

Trials where PLWH were not mentioned were presumed inclusive, as the absence of restrictive wording typically reflects more flexible and permissive protocol frameworks, allowing investigator discretion and favoring inclusion when no safety concerns are specified. Key policy milestones used to contextualize temporal trends included publication of the AFCR-HIV (November 2017), its adoption in NCI-US protocols (December 2021), and incorporation into CCTG generic protocol templates (August 2024).

### Statistical Analysis

People living with HIV eligibility status was summarized using descriptive statistics. Associations between PLWH eligibility and trial characteristics, such as cancer type (solid, hematologic, mixed), intervention type, trial phase, lead group (CCTG vs. non-CCTG) and industry support, were assessed using the Chi-square test. Univariable and multivariable logistic regression analyses were performed, with adjusted *p*-values reported for the multivariable models, and assessment of interaction between variables. Multivariable logistic regression models were then constructed to estimate adjusted odds ratios, accounting for the overlapping effects of industry sponsorship, immune checkpoint blockade therapy (ICB) and other covariates considered in the paper. Model results are reported as odds ratios with 95% confidence intervals, and statistical significance was assessed using two-sided *p*-values. The proportion of trials excluding PLWH versus inclusive of PLWH (eligible and not mentioned) was evaluated over time and categorized by trial activation period. A logistic regression model was used to explore the relationship between PLWH eligibility criteria and trial activation year, with time modeled as a continuous variable.

## 3. Results

Of 136 trials activated between 1999 and 2025 (Table 1), 83 (61%) were open to accrual, and 53 (39%) were in ongoing follow-up as of Spring 2025. The majority were phase III trials (n = 80, 61.5%), with solid tumors as the primary cancer type (n = 111, 81.6%). Most of the trials (n = 104, 76.4%) incorporated systemic therapy, either alone or in combination with surgery or radiation. ICB was part of 34 trials (25%). Overall, 78 (57.4%) trials received industry support, either direct financial contributions or in-kind support, most commonly the provision of study drugs. Most trials were led by groups outside CCTG (n = 82, 60.3%). Of the 136 trials analyzed, 51 (37.5%) were activated between 1999 and 2017, 64 (47.1%) between 2018 and 2023, and 21 (15.4%) in 2024–2025.

**Figure 1:**
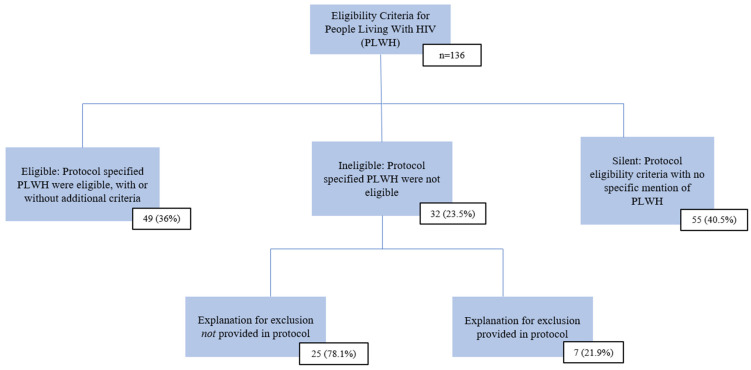
People living with HIV eligibility in Canadian Cancer Trials Group protocols.

Regarding PLWH eligibility, 49 protocols (36%) explicitly stated that PLWH were eligible. In 55 (40.5%), PLWH were not mentioned. Thirty-two protocols (23.5%) explicitly excluded PLWH. Among these, seven (21.9%) provided a rationale for exclusion (Figure 1).

The most often cited rationale for exclusion was potential pharmacokinetic interactions with ART and theoretical risks of increased toxicity, as seen in trials involving cediranib, olaparib, nivolumab, and triapine (see Table 2).

In the univariate analysis, trials evaluating low-risk interventions (cancer prevention, behavioral/lifestyle and cancer screening) were more likely to include PLWH than those using systemic therapies (93.8% vs. 71.2%, *p* = 0.008). PLWH were more likely to be included in later-phase trials (phase II/III and III) compared to early-phase trials (82% vs. 61%, *p* = 0.010). Trials including ICB had lower rates of PLWH eligibility than trials without ICB (53% vs. 84%, *p* < 0.001). Trials with industry support were more likely to exclude PLWH compared with non-industry-funded trials (37.2% vs. 5.2%, *p* < 0.001); 75 (96%) of the industry-supported trials included systemic therapy.

On multivariable analysis, only trials with industry support (*p* = 0.014) and those including ICB (*p* = 0.039) remained significantly more likely to exclude PLWH. In the univariable analysis, industry-supported trials had significantly lower odds of including PLWH (OR 0.09; 95% CI 0.03–0.32). After adjustment for ICB therapy and other covariates, the association remained significant, although attenuated (adjusted OR 0.16; 95% CI 0.04–0.68). Similarly, trials involving ICB had lower odds of including PLWH compared with trials without ICB (OR 0.21; 95% CI 0.09–0.49). This association persisted after adjustment for industry sponsorship and other covariates, with reduced magnitude (adjusted OR 0.35; 95% CI 0.13–0.95).

On multivariable analysis, there was no significant difference in PLWH eligibility based on cancer type (hematologic vs. solid, *p* = 0.952). Trials led by non-CCTG groups showed a non-significant trend toward greater inclusivity when compared to trials led by CCTG (*p* = 0.478). Among the trials opened prior to August 2024, when CCTG formally incorporated the AFCR-HIV guidelines, 85 of 115 (74%) protocols included (or did not mention) PLWH, compared to 19 of 21 (90.5%) of the later trials (*p* = 0.517) (Table 3). The Cochran–Armitage trend test logistic regression model with time as a continuous variable did not demonstrate sufficient evidence to support a trend towards inclusion of PLWH over time (*p* = 0.280).

### Specific Protocol Requirements for Eligible PLWH (Table 4)

In the 49 clinical trial protocols that explicitly included PLWH, 32 required participants to be on ART, 14 did not mention ART, and one trial excluded participants on ART. One allowed enrollment regardless of ART use but under specific conditions, including meeting CD4 count criteria. Another trial specified that PLWH could enroll regardless of ART (including no ART), provided there was no intention to initiate therapy or the regimen had been stable for at least four weeks with no intention to change the regimen within 12 weeks following enrollment. Potential ART interactions were addressed in 10/49 trials that included PLWH.

There were a variety of approaches to CD4 count among the 49 protocols. Some required minimum thresholds: ≥400 (n = 1), ≥350 (n = 5), ≥300 (n = 1), ≥200 (n = 4) and >200 (n = 1). Two acute leukemia trials acknowledged that T-cell counts may not be informative. In 35 protocols, CD4 count was not mentioned.

Viral load requirements were unspecified in 16 trials, while others required it to be undetectable with no further specifications (n = 9), undetectable within six months prior to enrollment (n = 20), or within 16 weeks prior to enrollment (n = 2). Only two trials specified a maximum of 25,000 IU/mL.

Most trials did not reference opportunistic infections requirements (n = 41); a few excluded individuals based on AIDS history (n = 7) or evidence of immunosuppression (n = 1). On antimicrobial prophylaxis, 47 trials were silent, one required Pneumocystis Jirovecii pneumonia prophylaxis and another excluded those needing prophylaxis for any opportunistic infections.

Additional PLWH eligibility language included: (1) PLWH are eligible as long as infection is adequately controlled in the opinion of the investigator; (2) not new diagnosis of HIV within 12 months prior to study registration; (3) medical conditions such as uncontrolled infection (including HIV), which, in the opinion of the treating physician, would make this protocol unreasonably hazardous for the patient; (4) patient must be healthy on the basis of HIV disease with high likelihood of near normal life span were it not for the cancer; and (5) HIV is sensitive to ART.

**Table 4:** HIV-specific inclusion criteria among trial protocols permitting enrollment of people living with HIV (n = 49).

Eligibility Domain	Criterion	Number of Trials, n (%)
**ART-related criterion**	Required participants on ART	32 (65.3)
	Did not mention ART status	14 (28.6)
	Explicitly excluded participants on ART	1 (2.0)
	Allowed enrollment regardless of ART use under specific conditions (e.g., meeting CD4 criteria)	1 (2.0)
	Allowed enrollment regardless of ART use	1 (2.0)
**ART-drug interactions**	Addressed potential ART–drug interactions	10 (20.4)
	No mention of ART–drug interactions	39 (79.6)
**CD4 count**	Not specified	35 (71.4)
	≥400 cells/µL	1 (2.0)
	≥350 cells/µL	5 (10.2)
	≥300 cells/µL	1 (2.0)
	≥200 cells/µL	4 (8.2)
	>200 cells/µL/not exclusionary	1 (2.0)
	CD4 count not considered informative	2 (4.1)
**HIV viral load**	Not specified	16 (32.7)
	Undetectable (no timing specified)	9 (18.4)
	Undetectable within 6 months before enrollment	20 (40.8)
	Undetectable within 16 weeks before enrollment	2 (4.1)
	≤25,000 IU/mL	2 (4.1)
**Opportunistic infections**	Not specified	41 (83.7)
	AIDS history excluded	7 (14.3)
	Evidence of immunosuppression excluded	1 (2.0)
**Antimicrobial prophylaxis**	Not specified	47 (95.9)
	PJP prophylaxis required	1 (2.0)
	Excluded if prophylaxis required	1 (2.0)

ART: Antiretroviral therapy.

Information on the actual enrollment of PLWH to Canadian Cancer Groups Trials was not a prespecified objective of the present study. To provide limited contextual insight, we conducted an exploratory search to identify trials in which PLWH were known to have enrolled. Between 2012 and 2025, ten participants with HIV were identified from electronic case report forms on trials for multiple myeloma, bladder, brain, breast, head and neck, and cervical cancers.

## 4. Discussion

Our analysis revealed variable compliance with contemporary HIV eligibility guidance across CCTG protocols. This was not unexpected, as until recently, there was no programmatic process in place to ensure systematic implementation of modernized criteria across all trials. In August 2024, CCTG incorporated revised protocol templates to explicitly address the inclusion of PLWH, which aligned eligibility language with AFCR-HIV recommendations and established a framework for greater consistency moving forward.

The persistence of HIV-related exclusions in some CCTG protocols highlights the incomplete translation of international eligibility modernization efforts into routine trial design. However, the overall exclusion rate in this analysis was lower than previously reported in US and international cohorts [17,18,19,20].

Most eligibility restrictions described in protocols were based on concerns about pharmacokinetic interactions or immune-related toxicity as perceived at the time of study conduct. Over time, data has emerged demonstrating the safety and feasibility of administering systemic therapy, including ICB, in well-controlled HIV. These findings reinforce the need to continually reassess categorical exclusions with evidence-based, context-specific criteria that reflect contemporary HIV management and evolving oncology standards and knowledge.

Before uniform incorporation into CCTG generic protocol templates in 2024, uptake had varied by therapy type and context. Notably, formal recommendations to include PLWH in cancer clinical trials were issued by the AFCR-HIV in 2017, with endorsement by the NCI-US in 2021. Before these milestones, inclusion decisions largely depended on individual investigator comfort and awareness and protocol-specific safety considerations.

Trials using ICB had significantly lower PLWH eligibility rates. These newer agents initially caused concern for potential risk of exposing opportunistic infections and immune reconstitution inflammatory syndrome, as well as the unknown effects of immune stimulatory agents and unclear efficacy in the context of HIV-associated alterations in the T-cell repertoire. There is now emerging data supporting the safety of ICB in PLWH, with no increase in infections or Immune Reconstitution Inflammatory Syndrome. Trials like DURVAST and CITN-12 showed similar toxicity profiles to HIV-negative patients, stable HIV parameters, and modest efficacy (17–25% response rates) [21,22,23,24,25]. However, more powerful prospective studies are lacking.

Our analysis showed that industry-supported trials were significantly more likely to exclude PLWH. Although most industry-supported trials involved systemic therapies, the association between industry support and exclusion persisted after adjustment for ICB and other covariates, indicating an independent association. This pattern may reflect cautious eligibility criteria commonly used in industry trials, shaped by safety considerations, regulatory requirements, and operational factors.

On univariable analysis, some trial characteristics were significantly associated with PLWH inclusion. There was a trend towards greater inclusion in later-phase trials compared to early-phase. The higher exclusion rate in early-phase studies may reflect greater uncertainty about safety, pharmacokinetics, drug–drug interactions with ART and focus on early efficacy signals. The greater inclusivity observed in later-phase trials may be driven by more robust safety data from earlier studies, increased external advocacy to broaden eligibility, and recognition of the need for trial populations to reflect real-world demographics. The trend towards higher inclusivity in non-systemic therapy trials suggests that perceived risks of systemic toxicity or drug–drug interactions remain a factor that could influence exclusion of PLWH from some cancer trials. However, the relationship between these variables and PLWH eligibility was not statistically significant on multivariable analysis. Although trials led by non-CCTG groups appeared more inclusive, this trend was not statistically significant and may be attributable to differences in trial phase distribution rather than institutional policies or practices.

Even though we did not observe a statistically significant trend toward greater inclusion of PLWH over time (Cochran–Armitage *p* = 0.280), an increase in inclusive trial design (74% to 90%, *p* = 0.517) was noted among CCTG trials since 2024, which is consistent with findings from other authors in US-based clinical trials [20]. This pattern appears encouraging but should be interpreted with caution, as no definitive conclusions regarding trends over time can be drawn.

The CTEP experience demonstrates that meaningful eligibility modernization depends on committed sponsors to implement AFCR-HIV guidelines across protocols [26]. Our findings reflect this principle and are likely generalizable to other cooperative groups with similar structures. However, generalizability may be limited in non-Canadian settings where regulatory frameworks and HIV management practices differ.

Eligibility requirements varied across trials, as described in Section 3, with respect to ART use, CD4 count thresholds, viral load restrictions, consideration of opportunistic infections, antimicrobial prophylaxis, and potential ART interactions. Some protocols allowed broad inclusion with minimal HIV-specific criteria, while other ‘eligible’ trials imposed stringent thresholds or exclusionary conditions, such as CD4 cut-offs > 350, contradicting the AFCR-HIV principle of minimizing thresholds. These findings highlight that nominal inclusion does not necessarily equate to meaningful access and underscore ongoing variability in the application of HIV-related eligibility criteria. This variability likely reflects the evolving evidence base regarding HIV management in oncology and the diversity of trial designs, interventions, and therapeutic contexts represented in our analysis.

Protocol eligibility restrictions are among several potential barriers limiting accrual of PLWH to clinical trials. The ten participants we know of who enrolled in CCTG trials are likely an underestimate of the total number enrolled. We were only able to screen trials where CCTG holds the database, and in an electronic format. Not all trials capture details of medical comorbidities—for example, studies of psychosocial interventions. Regardless, we suspect PLWH are under-represented compared to the population. In order to not risk inadvertently making patients identifiable, limited comments can be made about these small numbers. Further studies would be needed to understand whether, in practice, when inclusion of PLWH is permitted, they are invited to participate in clinical trials, and whether they choose to enroll.

### Limitations

This protocol-based review may underestimate true exclusion rates. We assumed that the absence of explicit exclusion criteria for PLWH equates to their inclusion. In practice, this may not hold true due to implicit bias, site-specific practices, or clinician discretion. Our analysis of temporal trends was limited by the relatively small number of trials activated in 2024–2025 (n = 21), reducing the statistical power to detect changes following the adoption of AFCR-HIV guidelines. The study could be considered as using the adoption of CCTG protocol templates as both intervention and outcome; more time and trials are needed to determine overall impact. Although we explored associations between trial characteristics and PLWH eligibility, these relationships are observational and may be confounded by unmeasured factors such as investigator attitudes, therapeutic context, or institutional policies. Details regarding HIV-related eligibility criteria were inconsistently reported, limiting our ability to analyze these requirements systematically. This analysis may be subject to selection bias, as it included only active or follow-up CCTG trials and excluded permanently closed studies, potentially underrepresenting historical exclusion practices. Additionally, because the review focused solely on CCTG protocols, findings may not reflect the broader Canadian cancer trial landscape, including those conducted independently or by industry sponsors.

## 5. Conclusions

Our analysis of 136 CCTG multi-centre trials revealed that although PLWH were eligible in most trials, nearly one in four still explicitly excluded them, often without providing a rationale. This exclusion was most common in early-phase and systemic therapy trials, where concerns around safety may be driving decisions. These findings matter to multiple stakeholders: clinicians seeking evidence-based treatment for HIV-positive cancer patients, drug developers aiming for regulatory approval based on inclusive data, and most importantly, patients who deserve access to potentially life-saving therapies.

CCTG’s adoption of revised protocol templates in 2024, aligned with AFCR-HIV recommendations, represents a step toward more equitable and evidence-based trial design. This proactive change encourages thoughtful eligibility criteria and promotes a cultural shift toward inclusion of PLWH in cancer research. Incorporation of AFCR-HIV recommendations as a regulatory standard could reduce unjustified exclusions and promote harmonization across trials. Sponsors should be required to provide justification for exclusion in clinical trial protocols. Future research should measure accrual of PLWH to clinical trials and explore other potentially reversible barriers to their participation. Ongoing safety and efficacy monitoring of emerging therapeutics, such as cellular therapy, in PLWH is essential as both HIV and cancer care continue to evolve.

## Figures and Tables

**Table 1:** Clinical trial characteristics.

Trials	n = 136 (%)
Phase I/II	41 (31.6)
Phase II/III	9 (6.9)
Phase III	80 (61.5)
Cohort	5 (3.7)
Screening	1 (0.7)
**Tumor type**	
Solid	111 (81.6)
Hematological	20 (14.7)
Mixed	5 (3.7)
**Lead group**	
Canadian Cancer Trials Group	54 (39.7)
Other	82 (60.3)
**Year activated**	
1999–2017	51 (37.5)
2018–2023	64 (47.1)
2024–2025	21 (15.4)
**Intervention type**	
Systemic therapy	86 (63.2)
Combination with systemic therapy	18 (13.2)
Combination without systemic therapy	1 (0.7)
Low-risk interventions ^a^	12 (8.8)
Radiation	12 (8.8)
Immunization	2 (1.5)
Surgery	5 (3.7)
**Trial status as of spring 2025**	
Open to accrual	83 (61)
Closed to accrual	53 (39)
**Industry funding**	
Yes	78 (57.4)
No	58 (42.6)
**Immune checkpoint blockade therapy**	
Yes	34 (25)
No	102 (75)

*^a^ Cancer prevention, behavioral/lifestyle and cancer screening.*

**Table 2:** Protocol-reported reasons for exclusion of people living with HIV (n = 7).

Exclusion Rationale	Number of Trials n (%)
Potential for pharmacokinetic interactions with triapine.	1 (14.3)
Potential for pharmacokinetic interactions with cediranib or olaparib. Also, increased risk of lethal infections when treated with marrow-suppressive therapy.	2 (28.6)
Lack of data regarding the effects of nivolumab on antiretroviral therapy, which had not been studied.	2 (28.6)
Unknown effects of ipilimumab in the setting of chronic viral infections.	1 (14.3)
Compromised immune function and the possibility of early death were perceived as potentially compromising study objectives.	1 (14.3)

**Table 3:** Associations between clinical trial characteristics and protocol ineligibility criteria for people living with HIV.

Clinical Trial Characteristics	PLWH Ineligible	PLWH Eligible and Not Mentioned	*p* Value *	
			*Univariable Analysis*	*Multivariable Analysis*
All trials (n = 136)	32 (23.5%)	104 (76.5%)	-	-
Trials *with* industry funding (n = 78)	29 (37.2%)	49 (62.8%)	<0.001	0.014
Trials *without* industry funding (n = 58)	3 (5.2%)	55 (94.8%)		
Trials *including* systemic therapy (n = 104)	30 (28.8%)	74 (71.2%)	0.008	0.802
Trials *not including* systemic therapy (n = 32)	2 (6.3%)	30 (93.8%)		
Trials *including* immune checkpoint blockade therapy (n = 34)	16 (47%)	18 (53%)	<0.001	0.039
Trials *not including* immune checkpoint blockade therapy (n = 102)	16 (16%)	86 (84%)		
Phase *I*/*II* trials (n = 41)	16 (39%)	25 (61%)	0.010	0.464
Phase *II*/*III*, *III* trials (n = 89)	16 (18%)	73 (82%)		
Trials *led by CCTG* (n = 54)	17 (31.5%)	37 (68.5%)	0.076	0.478
Trials led by *other groups* (n = 82)	15 (18.3%)	67 (81.7%)		
Trials activated *after* CCTG’s incorporation of AFCR-HIV recommendations, August 2024 (n = 21)	2 (9.5%)	19 (90.5%)	0.099	0.517
Trials activated *before* CCTG’s incorporation of AFCR-HIV recommendations, August 2024 (n = 115)	30 (26%)	85 (74%)		
Trials in *solid tumors only* (n = 111)	26 (23.4%)	85 (76.6%)	0.951	0.952
Trials in *hematology malignancies and mixed* (n = 25)	6 (24%)	19 (76%)		

CCTG: Canadian Cancer Trials Group; AFCR-HIV: American Society of Clinical Oncology Friends of Cancer Research-Eligibility Criteria for People Living With HIV; PLWH: people living with HIV; * Compares the proportion of PLWH classified as ineligible versus eligible across trials **with and without** each trial characteristic.

## Data Availability

The raw data supporting the conclusions of this article will be made available by the Canadian Cancer Trials Group upon reasonable request. Restrictions may apply. Contact datasharing@ctg.queensu.ca or see the Data Sharing Policy available at https://www.ctg.queensu.ca.
